# Influence of jump height on the game efficiency in elite volleyball players

**DOI:** 10.1038/s41598-023-35729-w

**Published:** 2023-06-01

**Authors:** Damian Pawlik, Dariusz Mroczek

**Affiliations:** Department of Human Motoric Bases, Wroclaw University of Health and Sport Sciences, Str. Paderewskiego 35, 51-612 Wrocław, Poland

**Keywords:** Risk factors, Musculoskeletal system

## Abstract

The aim of the study was to verify the impact of the jump height measured during the serve, attack and block on their effectiveness. The analysis of the literature and observations lead to the hypothesis that despite the similarities in men's and women's volleyball, the nature of the way of playing between women and men differs. The observation covered 39 male and 24 female elite volleyball players. We used a proprietary software tool, namely OpenCV to assess the jump height library. The analysis was performed on the jump serve height (SJH), attack jump height (AJH), and block jump height (BJH). The following analysis was performed to confirm the differences in the height of the jump and partially confirm the hypothesis. The main difference between men's and women's volleyball is how the serve, attack and block jump height affect winning or losing match sets. In male volleyball players, there is a decrease in the parameters of SJH, AJH and BJH in subsequent sets of matches, while in female volleyball players this relationship is reversed. For men, it seems reasonable to strive to increase the height of a jump to maximize effective offensive actions, as well as to maintain the developed performance throughout a match. In women's volleyball, it is worth using such training measures that allow players would achieve their maximum jumping abilities from the first set, and the main training goal should be focused on the technique and tactics of the whole team.

## Introduction

Volleyball is one of the world's most popular team sports, characterized by explosive movement patterns and agile and quick positioning. The result of a volleyball game depends on the optimal combination of motor and technical-tactical factors. It is believed that an elite volleyball player should be characterized by a significant body height, a high level of dexterity and muscular power, especially jumpiness^1–4^. Jumping together with the right tactics determines the effectiveness of an attack, serve or block^5–7^. Sattler et al.^8^ compared vertical jump heights of professional male and female volleyball players from Slovenian I and II Division. The results show significant differences between Division I (DI) and II (DII) and male and female players in each jump: squat jump (DI 40.8 ± 5.6 cm vs. DII 37.2 ± 4.1 cm; countermovement jump (DI 45.3 ± 4.9 cm vs DII 42.5 ± 5.2 cm); block jump (DI 49.1 ± 5.6 cm vs. DII 46.9 ± 6 cm); attack jump (64.4 ± 6.9 cm vs DII 60.7 ± 7.4 cm). The results are also confirmed in the Belgian, Spanish and Portuguese leagues^9–11^. Unfortunately, the studies mentioned above are based on comparisons of individual jumps (SJ, CMJ, AJ) performed in laboratory conditions and effectiveness during a game that was assessed later. This combination omits the jump performed during an action in a real game. Modern video-tracking technologies allow observer to compile the correct height of a jump performed during a particular serve, attack or block^12–14^.

In women's and men's volleyball, the importance of serving, attacking and blocking as skills determining the winning-losing status during a game is emphasized^1,5,15^. Nevertheless, numerous studies indicate differences between male and female volleyball^16–18^. Despite the higher net (2.43 m for men's volleyball; 2.24 m for woman's volleyball), attacks in men's volleyball are performed using greater force and with a faster pace than in women's volleyball^5,19^. A clear difference in the speed of the ball after an attack can be observed between the play style at different levels. Forthomme^9^ in Belgian two highest Belgian national Divisions observed the spiked ball's speed for men, varying between 61.2 km/h (38.6 m/s) and 112.3 km/h (69.78 m/s), while for women, the range was lower, averaging between 45.8 km/h (28.46 m/s) and 82.5 km/h (51.26 m/s). Seminati et al.^20^ verified that in Italian elite players from the second Italian Volleyball League, the mean ball speed was similar in different spike techniques for male was between 25.6 and 26.3 m/s and female 20.1 m/s to 22.2 m/s. In USA I Division NCAA, the ball speed for female volleyball players was between 15.5 to 16.1 m/s higher^14,21,22^. The Spanish female Superleague mean ball speed was recorded at 18.4 m/s^23^. In the young French National Team, the players' mean ball speed was 103.0 ± 7.0 km h^24^. The combination of higher parameters of jump height and ball speed in men, in relation to women proves the lower physical capacities due to gender.

The lower impact force of the ball caused by the lower physical capacities of female players determines a more significant number of attacks and fewer point serves, thus extending the duration of an action^6,25,26^. Male players have a significantly higher number of dynamic jump serves than women (52.2/69.9% vs. 16.6%)^4,27^. Also, the ball's speed is higher in men's case. Palao et al.^4^ also observed a lower ratio of aces and errors, which indicates taking more risks in the serve area.

The block is indicated as the third technical and tactical element affecting the winning-losing game status^28–31^. Palao et al.^6^ claim that the block differentiates elite men's volleyball teams. Male players take risks in the power jump serve to anticipate the ball's distribution more efficiently and perform technically correct blocking (block positioning) to neutralize the opponent's attack. The opponent attacking the ball chooses the attack's directions and form, and the setter's surprising distribution can make it challenging to perform a double or triple block. Many authors have studied the difference between single and double blocks, and the results showed that single blocks are less efficient^6,32,33^. A successful block is understood as a point block. However, the cooperation between the block and the defense (covering the appropriate part of the court) can also be described as an effective block because the block meets the assumptions and facilitates defense^34^. According to Sattler et al.^35^, the height of the jump in the block represents the potential to reduce the effectiveness of the opponent's attack. Although the authors indicate the difference between the number of effective blocks and winning or losing matches, no relationship was found between the height of the jump and the effectiveness of the block^34,36^.

All the differences can significantly affect the detailed characteristics of the game and the load on the players during a match. The height of a jump measured during the execution of a proper volleyball element, combined with the effectiveness of a serve, attack or block, is a more reliable indicator than measuring a single jump in laboratory conditions.

The above considerations prompted the authors to thoroughly analyze the relationship between the height of a jump and the effectiveness of a serve, attack and block in elite men's and women's volleyball matches. The authors also wanted to compare the effort characteristics of teams in 3:0 and 3:1 games.

In accordance, this study aim to verify the impact of the height of a jump measured during the execution of a serve, attack and block on their effectiveness. The analysis of the literature and observations lead to the hypothesis that despite the similarities in men's and women's volleyball, the nature of the way of playing between women and men differs.

### Study material

In the study, we observed volleyball players of the highest leagues. The observation covered 39 male volleyball players representing Australia, Poland, Argentina, Russia, Bulgaria, Cuba and Finland and 24 female volleyball players representing four clubs in the Polish Tauron Women's League (Highest level). Among the 24 female players, 12 were active representatives of Poland, the Czech Republic, Serbia, France and Puerto Rico (Table 1). Matches ending with a score of 3:0 in the case of male players and 3:0 and 3:1 in the case of women were used for the observations. The study was reviewed and approved by the Senate Committee on Research Ethics of the Wroclaw University of Health and Sport Sciences, Poland (no. 19/2016). The study was conducted according to the guidelines of the Declaration of Helsinki. No informed consent was needed to participate in the study because the publicly available video material of matches played in the Polish volleyball league was analyzed.

**Table 1:** Characteristics of the observed players.

Sex	Value	Body height (cm)	Body mass (kg)	BMI	Attack reach (cm)
Male	Mean	201.54	91.71	22.57	354.46
	SD	5.68	8.29	1.69	8.88
Female	Mean	185.78	74.51	21.51	309.44
	SD	6.23	7.08	1.30	11.61

### Study methods

**Figure 1:**
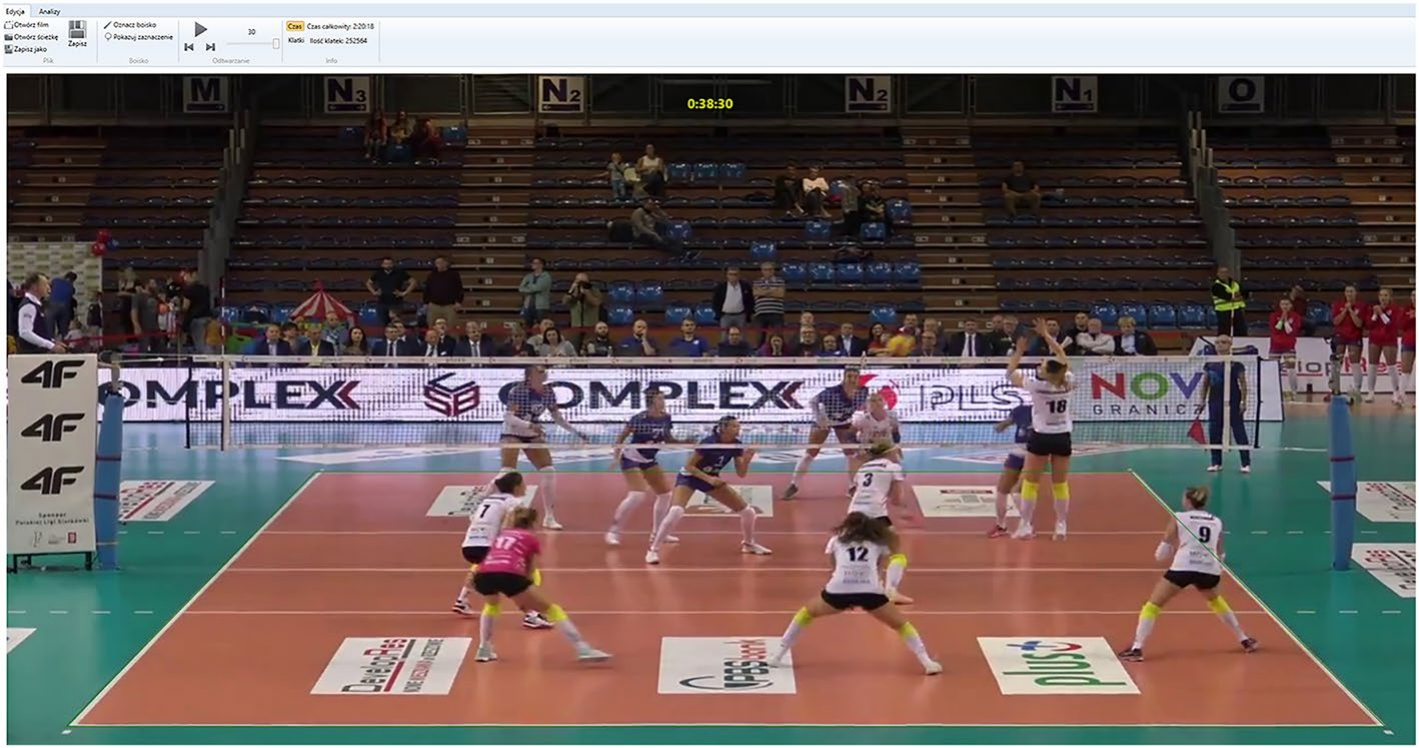
Software volleyball match analysis tool (authors property).

Matches that ended with the result of 3:0 and 3:1 were analyzed. The matches ending in 3:0 and 3:1 were selected for the analysis because the sets in these matches were played to 25 points. 3:2 matches were not taken into account because 5 sets are played to 15 (according to the rules of the game), which would significantly distort the analysis.This choice was supposed to make identifying the differences between teams in the same league easier. A proprietary software tool, namely OpenCV, was used to assess the jump height (available 28.06.2018r. on: https://opencv.org/) library (Fig. 1). All the calculations were performed in double precision (available 28.06.2018r. on: https://docs.microsoft.com). Two people participated in the collection of match data, who, after special training, performed video analysis using software. The analysis was performed on the jump serve height (SJH), attack jump height (AJH), and block jump height (BJH) (shift of a volleyball player's center of gravity in a vertical position).

Jump calculations were based on Halliday calculations^37^:

$${h}_{max}-h(t)=\frac{{gt}^{2}}{2}$$

where h stands for height, t is time, and g is the gravitational constant. We can determine a start and landing jump frame from a movie clip recording by the procedure described below. Jump duration is calculated as:

$$d={(f}_{landing}- {f}_{start})* {t}_{f}$$

where f stands for frame number and t f is the time of a single frame.

Jump ascent and descent times are equal, as they depend only on the gravitational constant.

Hence, descent time is $\frac{d}{2}$ and landing height is 0. Based on the above notes, we can create a formula:

$${h}_{max}=\frac{\mathrm{g}* [({(f}_{landing}- {f}_{start})*{t}_{f}{]}^{2} }{8}$$

### Effectiveness

All actions during a match were analyzed in terms of jump height and its effectiveness. The observed serves, attacks and blocks are marked accordingly with the symbolism and classification of the DataVolley and Volleystation statistical programs^5,38,39^. For statistical analysis, the effect of each serve, attack and block was assigned to the appropriate rank according to the value it presented^40,41^.

### Statistical analysis

Statistical analysis of the jump performance parameters was presented as an arithmetic mean and standard deviation of all the observed jumps. The interitem correlation coefficient (ICC) and Cronbach's alpha reliability coefficient (CA) were calculated to determine reliability between the jumps. Inter-variability for each test was measured by the coefficient of variation (CV) (Table 2). One-way analysis of variance (ANOVA) post hoc NIR test were used to analyze Jump Height and set number, the correlation between male and female and winning or losing status. The confidence interval (CI) was calculated for the determined mean values of each variable and aimed at marking limiting points within which there was a 95% probability that the sought population means of the variables could be reduced. All coefficients at a level of 95% (*p* < 0.05) were considered significant. The Spearman's rank correlation was calculated to determine correlations between the height of the jumps in serve, spike and block and their efficiency in the game. Data were analyzed using the software programs Statistica 13.3 [StatSoft (Europe) GmbH, Hamburg, Germany] and for calculation of ICC the software program SPSS statistics 25 (SPSS Inc., Chicago, IL, USA). Significance level was set at *p* < 0.05.

**Table 2:** The height of a jump during a serve, attack and block in men's and women's teams.

Variable	Sex	Mean (cm)	SD	ICC	CA	CV	Reliance − 95%	Reliance 95%	*p*-value
Serve height	M	62.91	19.60	0.89	0.89	0.15	61.06	64.77	
	F	38.33	14.80	0.92	0.92	0.22	36.60	40.06	0.001*
Spike height	M	70.31	18.71	0.87	0.87	0.38	68.81	71.81	
	F	46.40	14.22	0.89	0.89	0.21	45.08	47.71	0.001*
Block height	M	53.20	19.77	0.38	0.38	0.29	52.00	54.41	
	F	41.62	14.23	0.85	0.85	0.24	40.11	43.13	0.001*

*M* male, *F* female, *SD* standard deviation, *ICC* intraclass correlation, *CA* Cronbach's alpha reliability coefficient, *CV* coefficient of variation.

*Statistically significant at *p* < 0.05.

## Results

Table 2 contains values for jump height in serve, attack and block and a comparison between men and women. The expected statistically significant difference is in favor of men. The following analysis was performed to confirm the differences in the height of the jump and partially confirm the hypothesis.

**Figure 2:**
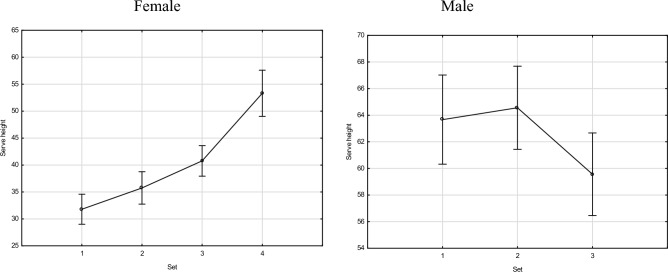
Female and male serve height (in cm) during sets.

The serve jump high increasing trends were observed in women. In each subsequent set, the female players jumped higher during the serve than in the previous one. The height of the jump during the serve in sets 3 and 4 was statistically significantly different from the height of the jump in sets 1 and 2 (*p* < 0.05). In men in set 3, statistically significant differences were observed, but the jump height was lower than in sets 1 and 2 (Fig. 2, Table 3).

**Table 3:** Differences between the height of a serve jump in individual sets.

Sex	Set	1	2	3	4
Mean (cm)		64.10	64.99	59.84	
M	1		0.701	0.067	
M	2	0.701		0.022*	
M	3	0.067	0.022*		
Mean (cm)		31.80	35.75	40.77	53.31
F	1		0.059	0.000*	0.000*
F	2	0.059		0.017*	0.000*
F	3	0.000*	0.017*		0.000*
F	4	0.000*	0.000*	0.000*	

*M* male, *F* female.

*Statistically significant at *p* < 0.05.

**Figure 3:**
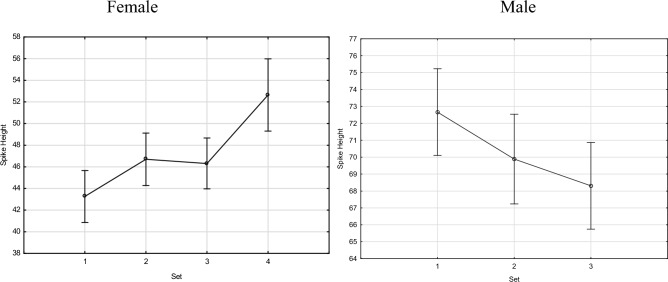
Female and male spike height (in cm) during sets.

Similar observations occurred in the height of an attack jump. In the case of women, increasing trends were observed, and in set 4 they differed statistically significantly at the level of *p* < 0.05. In the case of men, the trends were the opposite. In each subsequent set, the jump height values decreased. Statistically significant differences were observed between sets 1 and 3 (Fig. 3, Table 4).

**Table 4:** Female and male spike height during sets.

Sex	Set	1	2	3	4
Mean		72.66	69.90	68.35	
M	1		0.142	0.020*	
M	2	0.142		0.412	
M	3	0.020*	0.412		
Mean		43.36	46.53	46.03	52.77
F	1		0.070	0.120	0.000*
F	2	0.070		0.770	0.003*
F	3	0.120	0.770		0.001*
F	4	0.000*	0.003*	0.001*	

*M* male, *F* female.

*Statistically significant at *p* < 0.05.

**Figure 4:**
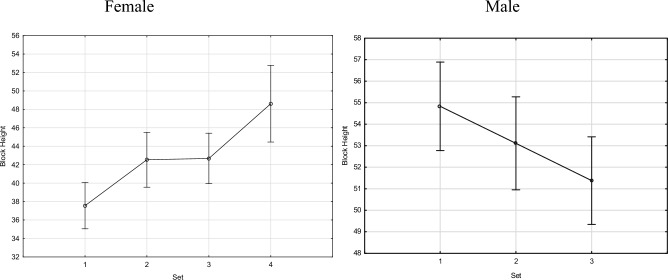
Female and male block height (in cm) during sets.

The distribution of jump height and block results in subsequent sets is identical to the other actions (serve and spike). Increasing jump scores were observed in women and decreasing in men (Fig. 4). Women differed statistically significantly in sets 1 and 4, while in the case of men set 3 differed considerably in relation to set 1 (Table 5).

**Table 5:** Block height and set.

Sex	Set	1	2	3	4
Mean (cm)		54.93	53.26	51.46	
M	1		0.272	0.019*	
M	2	0.272		0.232	
M	3	0.019*	0.232		
Mean (cm)		37.52	42.44	42.84	48.45
F	1		0.013*	0.005*	0.000*
F	2	0.013*		0.850	0.021*
F	3	0.005*	0.850		0.027*
F	4	0.000*	0.021*	0.027*	

*M* male, *F* female.

*Statistically significant at *p* < 0.05.

As a result of the verification of the study goal and hypothesis, the measured parameters of the jump height were analyzed, and divided into won and lost matches. In the case of women, a statistically significant difference was observed only in the case of attack height. Surprisingly, both attack and block heights were the opposite of the action's effectiveness. During the serve, jump height trends for teams that won and lost matches were similar for men and women. Teams that jumped higher during the serve generally won their matches. In the case of men, the teams that won their matches were characterized by higher jump height values during the serve, attack and block than the teams that lost the observed matches. In addition, the jump height during the serve and block was statistically significant at *p* < 0.05 (Table 6).

**Table 6:** Comparison of winning and losing teams.

Variable	Sex	Status	Mean	SD	− 95%	+ 95%	*p*-value
Serve height	M	W	65.37	19.17	62.95	67.79	
Serve height	M	L	59.69	19.75	56.83	62.55	0.003*
Serve height	F	W	39.15	16.95	36.36	41.95	
Serve height	F	L	37.45	12.20	35.45	39.52	0.289
Spike height	M	W	70.81	19.33	68.65	72.97	
Spike height	M	L	69.78	18.06	67.70	71.86	0.501
Spike height	F	W	44.36	12.64	42.74	45.98	
Spike height	F	L	48.60	15.48	46.53	50.67	0.001*
Block height	M	W	54.53	20.12	52.86	56.21	
Block height	M	L	51.66	19.27	49.93	53.39	0.020*
Block height	F	W	40.65	12.76	38.74	42.56	
Block height	F	L	42.60	15.57	40.25	44.96	0.191

*M* male, *F* female.

*Statistically significant at *p* < 0.05.

In order to achieve the aim of the study, a comparison of the relationship between the height of the jump and the effect of the serve, attack and block was made. In the case of women, a surprising statistically significant result is the negative correlation between the jump height and the block's effectiveness. A positive statistically significant relationship occurred in the height of a jump and block efficiency in men. In the case of both women and men during the attack and serve, no direct relationship with the height of the jump was observed. In addition, as far as the relationships between attack and serve effectiveness in men are concerned, a negative correlation between attack and block effectiveness and a negative correlation between block and attack effectiveness were observed (Table 7).

**Table 7:** Correlation between jump height and serve, spike and block efficiency.

Value	Sex	Serve height	Es	Spike height	Ea	Block height	Eb
Serve height	F	1.00	− 0.07	0.32*	− 0.05	0.22*	− 0.16*
Es	F	− 0.07	1.00	− 0.06	− 0.05	0.02	0.07
Spike height	F	0.32*	− 0.06	1.00	− 0.00	0.19*	− 0.08
Ea	F	− 0.05	− 0.05	− 0.00	1.00	0.05	− 0.02
Block height	F	0.22*	0.02	0.19*	0.05	1.00	− 0.12*
Eb	F	− 0.16*	0.07	− 0.08	− 0.02	− 0.12*	1.00
Serve height	M	1.00	− 0.04	− 0.03	− 0.06	0.08	− 0.04
Es	M	− 0.04	1.00	0.08	0.25*	0.04	0.02
Spike height	M	− 0.03	0.08	1.00	0.02	0.07	0.11*
Ea	M	− 0.06	0.25	0.02	1.00	− 0.06	− 0.09*
Block height	M	0.08	0.04	0.07	− 0.06	1.00	0.08*
Eb	M	− 0.04	0.02	0.11	− 0.09*	0.08*	1.00

*Es* serve efficiency, *Ea* spike efficiency, *Eb* block efficiency, *M* male, *F* female.

*Statistically significant at *p* < 0.05.

## Discussion

The obtained results confirm the hypothesis that despite the suggested similarities, the nature of match effort in volleyball between men and women differs significantly^16–19^. The main difference between men's and women's volleyball is how the serve, attack and block affect winning or losing. Referring to the observations of Palao et al.^4^, men more often take risks during the serve because a stronger serve performed with a higher jump increases the chance of scoring a point or making it more difficult for the opponent to hit the ball. Receiving the ball more than 3 m from the net or on the side of the court facilitates reading the play of the setter and effective blocking. Hence, the only direct correlation between jump height and block efficiency was observed. Our study confirms that a higher jump height in relation to serve and block positively affects the final status of a match in men's volleyball. When it comes to winning or losing a match in women's volleyball, the only and surprisingly negative correlation was observed during the attack. Such results suggest a more technical nature of the game than in the case of men. With a less risky serve (only about 16.6% of jump serves) and a lower pace of action, the technique of performing a given element and the tactics of the game most likely have a more significant impact on the effectiveness of the players^4,27^.

An interesting observation is the analysis of the height of a jump during the subsequent sets of a match. Male volleyball players, whose jump height in each element is much higher than women's, show the best sports performance in set 1. Each subsequent set is characterized by an apparent decrease in the observed jump height during the serve, attack and block (Table 1). Such a reduction in the jump height in all the elements is most likely the cause of increasing fatigue. From the first set of a match, men use their maximum motor skills to score a point. Starting from taking risks in the serve, through a high block, to the height of an attack, which in this case was not statistically significant between the teams that won and lost matches. However, compared to women, it was about 20 cm higher.

Female volleyball players jumped the lowest in the first set. In the second, third and fourth sets, the height of the jump was significantly higher. This may be the reason for poor preparation for the match or less importance of speed and strength abilities during the course of the observed matches. The analysis of the play style leads to the conclusion that women do not use their motor abilities, especially jump height, to control the game. Their main advantage is mostly the technique and quality of serves, attacks or blocks, with less risk. And more exchanges during an action necessitate more jumps. Such observations clearly differentiate the nature of the game, indicating strength and speed parameters in men and endurance and technical parameters in women, which affect success in a volleyball match.

Current analyses of jump height in relation to the result of matches and sets are based on laboratory measurements of squat jump (SJ), countermovement jump (CMJ) and their derivatives (Block Jump—BJ, Spike Jump—AJ). Jump height scores are measured in isolated conditions, which is not the case during a championship match. With the available measuring apparatus, it becomes possible to compare a jump during a particular action's performance with the effect of an attack, serve or block. Only such a comparison gave an accurate picture of the impact of the height of a jump on the effectiveness of the observed actions. Surprisingly, making such a list did not provide the expected results. Only the efficiency of the block, which is not directly dependent on the player, was related to the height of a jump in both women and men. The lack of a direct correlation between the effectiveness and the height of a jump in the attack and serve, as well as a statistically significant relationship between the height of a jump and winning a set and a match, prove that the height of a jump is a crucial parameter supporting the status of winning-losing in men's volleyball, which numerous authors confirm^1,5,9–11,15^.

In women's volleyball, the impact of jump height is not as significant as in men's volleyball. The lack of expected relationships, lower dynamics of hitting the ball and prolonged actions identify women's volleyball as a more technical game. The above studies, however, are a certain generalization of match effort in volleyball as a discipline in the case of women and men. Therefore, the above observations require confirmation in further studies. The dynamically developing approach of coaches to the motor preparation of women in volleyball blurs its actual character, striving, as in the case of men, for strength and speed preparation. However, it should be remembered that women's motor predispositions are different from men's. Therefore, training methods and means individually adapted to women's gender and capabilities should be used to maximize training effects.

## Conclusion

The above study confirms the posed hypothesis. Despite the similarities in the meaning of the serve, attack and block on the status in volleyball, the match effort of women and men differs significantly. The significant relationship between the height of a jump during the serve and the block had a significant relationship with the winning-losing status only in the case of men. For men, it seems reasonable to strive to increase the height of a jump to maximize effective offensive actions, as well as to maintain the developed performance throughout a match. In women's volleyball, it is worth using such training measures that allow players would achieve their maximum jumping abilities from the first set, and the main training goal should be focused on the technique and tactics of the whole team. On the other hand, observing the current women's matches, it is worth looking for players characterized by outstanding natural jumpiness and dynamics during recruitment and selection.

## Supplementary Information

Supplementary Information 1.

Supplementary Information 2.

Supplementary Information 3.

## Data Availability

The datasets used and/or analysed during the current study available from the corresponding author on reasonable request. All data generated or analysed during this study are included in this published article [and its supplementary information files].
